# Exploring the effects of digital technologies in health financing for universal health coverage: a synthesis of country experiences and lessons

**DOI:** 10.1093/oodh/oqae016

**Published:** 2024-06-26

**Authors:** Maarten Oranje, Inke Mathauer

**Affiliations:** WHO Consultant, Department of Health Financing and Economics, World Health Organization, 1211 Geneva, Switzerland; Department of Health Financing and Economics, World Health Organization, 1211 Geneva, Switzerland

**Keywords:** digital technologies, machine learning, health financing, health insurance, revenue raising, pooling, purchasing, universal health coverage

## Abstract

The use of digital technologies (DTs) for health financing receives increased attention in policy, practice and research. However, not much robust evidence exists on their effects on the intermediate and final universal health coverage objectives. This paper seeks to contribute to evidence gathering, by synthesizing the findings from nine country case studies which covered diverse applications of DTs and identified their effects on health financing and universal health coverage objectives. This paper also draws on review papers on the use of DTs for health financing. Our synthesis reveals that DTs can support and simplify health financing tasks and thus contribute to enhanced efficiency and transparency and more equitable resource distribution. If well designed, DTs can help overcome challenges inherent in paper-based data systems and enable otherwise hardly implementable policy options, especially options that rely on near real-time exchange of data. Yet, the studies also point to various risks. Caution is for instance required when the use of DTs enhances inequities between population groups due to various digital divides. The findings point to a number of policy orientations. There is need to include the application of DTs for health financing in national digital health strategies and to develop health financing specific guidance and regulation. This is for instance necessary to avoid that DTs negatively affect financial protection. Clear stipulations related to equity will serve to ensure that positive effects accrue to vulnerable population groups. The future research agenda calls for more and methodologically robust evidence generation with a clear universal health coverage orientation.

## INTRODUCTION

As a subset of the much broader domain of digital health interventions (DHIs) [1], the use of digital technologies (DTs) for health financing is receiving increased attention in policy, practice and research [2–5]. DTs are understood here as electronic tools, systems and devices that generate, store, process or transmit data [6]. Among others, this includes database and data analysis technologies, mobile phone applications, webpage interaction platforms, digital payments, blockchain, big data analytics and artificial intelligence (AI) including machine learning (ML) [7]. Not much robust evidence exists on the effects of applications of such DTs on health financing functions and their contribution to the intermediate universal health coverage (UHC) objectives (equity in resource distribution, efficiency, transparency and accountability) as well as the final UHC goals (equitable access, quality of care and universal financial protection) [2, 5]. The small body of existing literature focuses primarily on mobile phone technologies, most notably mobile payments and mobile health wallets [5].

Health financing is a core part of any health system, critical for accelerating progress toward UHC. Three health financing functions are commonly distinguished [8, 9] as follows.

- **Revenue raising** is the process of raising money to pay health system costs, inter alia through taxation and health insurance contributions. This includes policy decisions and tasks related to the sources and levels of contributions, and the mechanisms to collect these funds.
- **Pooling** is the accumulation and aggregation of prepaid funds, so that the financial risk of having to pay for health care is shared by all members of the pool. This includes decisions and tasks related to targeting and identification of population groups for subsidized health coverage as well as risk adjustment or cross-subsidization across risk pools.
- **Purchasing** is the process of allocating these prepaid and pooled funds from purchasers to health service providers. This includes decisions and tasks related to selection and contracting of providers, the provider payment methods and rates. Closely related to purchasing is benefits design, which concerns decisions on service and cost coverage, cost-sharing rates and exemptions.

**Figure 1:**
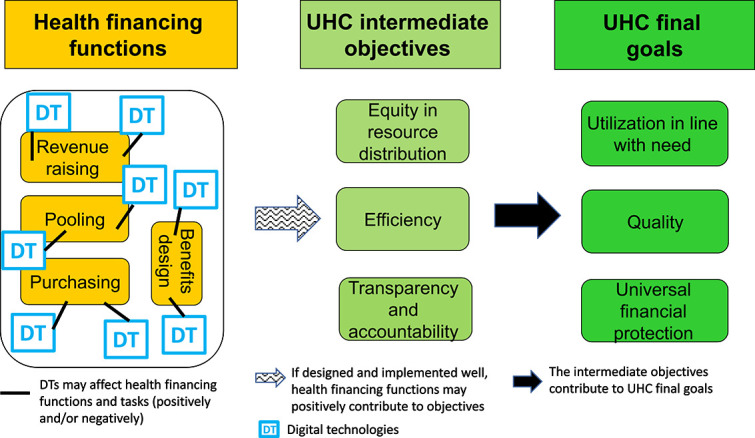
Linkage between digital technologies and health financing functions and potential effects on the intermediate and final UHC objectives (Source: (10))

The introduction of DTs can contribute to each of the health financing functions positively or negatively, depending on design features, implementation practice and contextual factors. Figure 1 shows the entry point of DTs, i.e. they operate at the level of the health financing functions and tasks and affect these and ultimately the intermediate and final UHC objectives [10].

The objective of this review paper is to contribute to evidence gathering regarding the effects of the use of DTs for health financing and to provide lessons and policy orientations in order to achieve positive effects on UHC objectives, as well as to inform the future research agenda.

## MATERIALS AND METHODS

This paper synthesizes findings from nine country case studies [11–17], while equally drawing on review papers on the use of DTs for health financing [2–5], which were all published in the last 5 years. The country case studies covered Estonia, North Macedonia, the Republic of Korea, Indonesia, the Philippines, Cambodia, India, Rwanda and Kenya, and focused on diverse applications of DTs for tasks in relation to health insurance or health coverage schemes.

The country cases were purposively selected for their relevant and advanced practice within their respective regions, representing a range of high-income, middle-income and low-income countries and applications across the three health financing functions, and for their potential to generate lessons for other countries. We use secondary data, i.e. the reports from the country case studies (as outlined further below). We applied thematic analysis along a WHO guide for assessing digital technologies in health financing that serves to identify their effects on health financing and UHC objectives [10]. For each health financing function, this guide provides a list of key questions relating to desirable health financing attributes and/or UHC objectives that help identify potential positive effects (benefits) as well as negative effects and risks. Likewise, the guide provides a list of indicators in relation to the guiding questions to measure or get an indication of change in the desirable health financing attributes and/or the UHC objectives. The desirable HF attributes are a list of 19 benchmarks, which are a crystallization of what matters in health financing in order to make progress to UHC [9].

The country case studies used the same (final draft version of the) WHO guide [10]. (An inductive approach was followed, whereby the draft of this guide was applied and findings from the country case studies were then fed back into the further development and finalization of this document.) The case studies were based on primary data collection and/or secondary data analysis as well as document review (with the exception of an entirely desk-based study for India, Rwanda and Cambodia) and were undertaken between 2021 and 2023.

Table 1 presents an overview of the nine countries, the health financing schemes and DTs assessed, and the health financing functions that were supported by these DTs. We have also categorized the DTs for health financing along the ‘Classification of digital health interventions’ [1] and provide the respective categories in square brackets.

**Table 1:** Overview of the country cases, the DTs assessed and the health financing functions supported

**Country**	**Health financing scheme**	**Digital technologies applied and assessed** **[categorization according to the classification of digital health interventions]**	**Health financing functions supported**
**Estonia**	Estonian Health Insurance Fund *[national health insurance]*	Digital claims management (*e-channel*), automated claim controls, ML for claims review, automated contract setting and monitoring [3.5.2; 4.1.4] Digital prescription system [2.9.1] and automated calculation and aggregation of cost-sharing amounts [1.7.1; 2.11.1].	Purchasing
**North Macedonia**	Health Insurance Fund of North Macedonia *[national health insurance]*	Digital claims management, digital contracts (*e-contracting*) [3.5.2], digital prescriptions (*e-prescriptions*) [2.9.1]	Purchasing
**Republic of Korea**	National Health Insurance Service *[national health insurance]*	Digital claims management, automated claims controls [3.5.2], patient web portal and mobile phone application [1.4.1], interoperable member management system for imposition of contributions [3.5.8]	Revenue raising, pooling, purchasing
**Indonesia**	Jaminan Kesehatan Nasional *[national health insurance]*	Mobile phone application for enrolment, revenue collection [1.7; 3.5.1] and patient information provision [1.4.1] (*Mobile JKN*)	Revenue raising, purchasing
**Philippines**	PhilHealth *[national health insurance]*	ML for fraud detection in claims [3.5.2; 4.1.4]	Purchasing
**Cambodia**	Health Equity Fund *[non-contributory health coverage scheme for poor and vulnerable households within a health financing system of multiple pools]*	Use of tablets, digital photographs and a web-based (interoperable) data system for beneficiary management by the purchaser [3.5.1] and at provider level [2.11.1]	Pooling
**India**	Ayushman Bharat Pradhan Mantri Jan Arogya Yojana *[non-contributory health coverage scheme for poor and vulnerable households, within a health financing system of multiple pools]*	Web-based data system to support patient data management, smartcards (using fingerprints, digital photographs, iris scans) [2.11.1], algorithm for identity confirmation [3.5.1]	Pooling
**Rwanda**	Community Based Health Insurance *[national health insurance for people in the informal economy, within a health financing system of multiple pools]*	Web-based (interoperable) data system for beneficiary management by the purchaser [3.5.1] and at provider level [2.11.1], web portal for mobile contribution payments [1.7]	Revenue raising, pooling
**Kenya**	National Health Insurance Fund *[national health insurance, within a health financing system of multiple pools]*	Digital claims management, [3.5.2], mobile phone application for enrolment and contribution payments [3.5.1; 1.7]	Revenue raising, purchasing

Source: Compiled by authors, based on [11–17].

All the country case studies focus on public health insurance or explicit health coverage schemes and the associated health financing tasks. While this facilitates the synthesis of the country cases, we recognize that DT applications for other health financing arrangements, such as through the government health budget are not included here, while applications for private/commercial health insurance only receive limited attention. Nor does this review cover the applications of DTs in domains directly adjacent to the health financing functions, such as in public financial management, or the management of financial flows within health facilities (which all arguably indirectly also affect health financing).

## RESULTS

The findings from the country case studies are presented along the three core health financing functions: revenue raising, pooling and purchasing (including benefits design). We provide short illustrations of specific issues in country case boxes.

### Revenue raising

One use case of DTs for revenue raising is to support the ‘determination of contribution rates’ of households, with the idea of ensuring fair financing [9], i.e. progressive or at least proportional payments for health. Enhanced interoperability tools allow to connect and cross-validate beneficiary data across different information management systems, such as tax databases, pension databases, social security registries, etc. For instance, in Rwanda, an application programming interface (API) connects the health insurance membership management system to a system that contains all relevant socio-economic household data (the so-called ‘Ubudehe’ system), making it possible to determine the applicable contribution for a household depending on its socio-economic level. In the Republic of Korea, the data system of the National Health Insurance Service (NHIS) is connected to the data bases and systems of more than 30 other organizations, facilitating eligibility management and the exchange of data required to compute health insurance contributions. For the NHIS, this enables near real-time checking of the employment status. It also makes it possible to compute the contribution amounts of self-employed people by drawing upon a large number of income and asset-related variables, which would be cumbersome and rather time-consuming, if not impossible, without the use of interoperable data bases. At the same time, interoperability can also pose risks as insufficient protection of personal data could lead to breaches of privacy and misuse of data.

Other revenue raising tasks that are frequently supported by DTs are ‘member registration and the collection of health insurance contributions’, particularly for the informal sector population [5], as is for instance the case in Indonesia (see Box 1), Kenya (see Box 2) and Rwanda (see Box 3). Mobile phone and web portal applications intend to reduce the time, effort, transportation and other costs incurred by members to make payments, while the health coverage or health insurance agency benefits from lower operational and administrative costs. Often, the mobile phone or web portal applications also serve to perform other tasks, such as ‘enrolment and enrolment reminders’, as well as ‘personal tracking of enrolment and payment status’.

While lowering the threshold for active engagement, and likely enhancing efficiency, there is also a risk that using mobile phone applications for these processes puts certain groups at a disadvantage, due to digital divides within the population [2, 5]. In Indonesia, for instance, when ‘Mobile JKN’, a mobile application for insurance enrolment and access to information, was introduced, the uptake by rural and poorer population groups was lagging behind. A lack of skills, information and understanding of the process among the target population were among the challenges observed, together with limited smartphone ownership, network disruptions and downloading problems related to the size of the application [18]. Since such digital divides and challenges are typically experienced more frequently by people in lower income quintiles (as is true for the use of other DTs), correlating with lower educational levels, they may very well exacerbate inequities in access and in financial protection. Similarly, in Kenya, while access to mobile money and the use of mobile applications for membership renewal is saving both time and costs for users—leading to an increased probability of insurance (NHIF) enrolment among rural dwellers [19]—there are still limitations to including the informal sector population.

**COUNTRY BOX 1: INDONESIA**

Indonesia’s Mobile JKN is a mobile application (app) with multiple features, inter alia allowing people to enroll as members for the national health insurance (Jaminan Kesehatan Nasional, JKN), register for auto-debit payment of insurance contributions, request deferral of contribution payments, register with primary health care providers, make doctor appointments and file complaints. The application was launched in 2017 and had been downloaded 11 million times by late 2020. Its main objective is to improve access to health services, while enabling administrative and operational cost savings as well as increased collection of insurance contributions. In its initial form, Mobile JKN faced several challenges, including technical caveats, a lack of awareness and digital illiteracy among intended users, leading to unequal access. During and in the aftermath of the COVID-19 pandemic, several functions have been added to the app and a new, more user-friendly version has been introduced. Since 2022, a surge has been observed in the number of active Mobile JKN users, with the number of downloads increasing to more than 21 million and a better representation of low-income groups [15].

**COUNTRY BOX 2: KENYA**

In Kenya, the National Health Insurance Fund (NHIF) no longer collects any cash, but instead receives all contributions from members and their employers (for those in the formal sector) in the form of digital payments. Member contributions can either be made through digital bank transfers or through mobile money transfers (using platforms such as M-PESA or Airtel money), saving time, effort and travel costs for users, while another benefit is the reduced administrative burden for the NHIF. The introduction of the My NHIF App in 2019 allowed individuals to register with the NHIF *and* to pay their NHIF contributions; this holds promise to improve the collection of contributions for NHIF [17].

**Figure 2:**
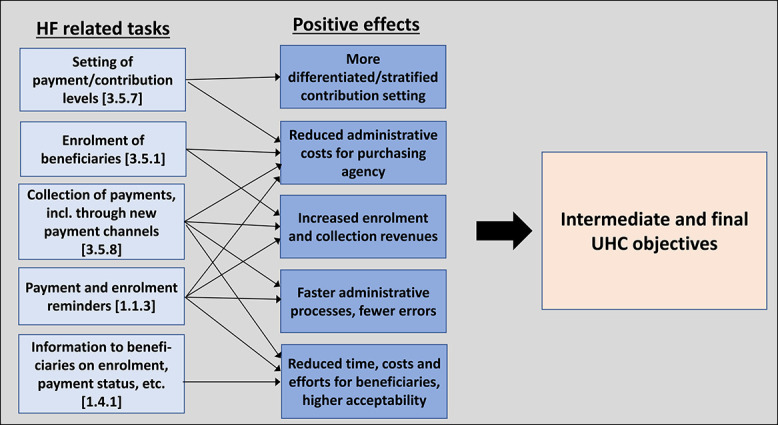
Revenue raising tasks supported by DTs, linked to potential positive effects and UHC objectives

Figure 2 summarizes the main tasks under the revenue raising function found to be supported by DTs and the potential positive effects, which contribute to the intermediate and final UHC objectives.

### Pooling

Various pooling related tasks are found to be supported by DTs. One key area is the ‘targeting and identification’ (including identity confirmation) of beneficiaries to be included in (subsidized) health coverage schemes. Where data collection on household assets and income to determine eligibility status for subsidized health insurance takes place through face-to-face interactions at the community level, as in Cambodia, the use of tablets or smartphone applications as well as digital photographs and biometric data is an important first step to digitizing data, thus helping to enhance the speed and accuracy of data processing [11]. Furthermore, digitalized web-based information management systems enable faster and more efficient identification processes, as household composition data (e.g. birth and death registration data, employment status) can be updated in near real-time as opposed to paper-based or offline information systems. DTs also improve targeting accuracy (i.e. the reduction of exclusion and inclusion errors). In Cambodia, the ID Poor system is interoperable with other government databases which register the poverty or otherwise vulnerable status of households. In Rwanda, the ‘Local Administrative Entities Development Agency Monitoring and Evaluation Information System’ (LODA MEIS), commonly referred to as the ‘Ubudehe’ system, is used to determine and register the socioeconomic status of the entire population in order to identify those eligible for full or partial subsidization, while interoperability with the national ID system helps to confirm the identity of beneficiaries [11].

As existing poverty data from multiple sources are exchanged, re-used and triangulated, unnecessary duplication of data collection and data management is avoided, reducing administrative costs in the long-term. Both in Rwanda and in Cambodia, interoperable and integrated databases across multiple government sectors are in place, informing the targeting process of the poor for diverse interventions and thereby increasing efficiency [11]. In India, an algorithm is applied to check and confirm an individual’s identity: when there are differences in spelling of names and/or geographical locations between the beneficiary database of the national health insurance and the digital identity card presented by a patient, the algorithm computes a confidence score expressing the likelihood that this concerns the same person [11].

DTs and specifically applications of ML have the potential to further support the automation of targeting and identification processes, by revealing more granular insights as to which households require (financial) assistance, contributing to more effective targeting and identification strategies and population coverage policies, ultimately also improving access and financial protection [3].

**COUNTRY BOX 3: RWANDA**

In Rwanda, determining the eligibility of beneficiaries for subsidized health coverage under the Community Based Health Insurance (CBHI) is greatly facilitated by interoperability and automated data exchange between several government information systems. The ‘Mutuelles Members Management System’ (3MS), introduced in 2018, is linked to the ‘Ubudehe’ system, which is a nationwide, web-based database containing frequently updated household composition and socio-economic data. This allows to identify eligible households for fully or partially subsidized coverage, and to determine the different contribution rates. The 3MS system is also connected with an e-Government portal for mobile payments. This enables health providers to check that patients are registered CBHI members and have paid their contributions, which is a simpler process than checking against the previous membership cards. Moreover, since 2019 the Ubudehe system is connected to the national identification system (NIDA). As such, the identity of individuals can easily be confirmed at health provider level and changes in their personal situation only need to be processed once within the overall information management system. Beyond important efficiency gains (lower costs, reduced time and fewer data registration errors), it is difficult to imagine how stratification of CBHI contributions could have been implemented effectively and reliably without a data architecture and near real-time updating of information [11].

Another pooling-related task that can be supported by DTs is ‘risk scoring’. By identifying specific patient attributes or other determinants of health, for instance, ML models support the ‘prediction of health expenditure or health needs’ of population groups or individuals more accurately. Besides allowing for better planning, these insights can be used to develop risk adjustment/equalization formulas that better reflect future health expenditure, so as to realize a more equitable allocation of resources across multiple pools [3]. As risk adjustment calculations require information on a range of variables, such as on health service utilization, enrolment and claims data as well as socio-economic and demographic data of different population groups, interoperability between the information systems of several social (health) protection or other government programs is again critical. Through the use of APIs, for instance, data can be exchanged in (near) real-time, compared and combined, contributing to enhanced cross-subsidization or risk adjustment mechanisms, and in the long run potentially even facilitating pool-merging [2]*.*

At the same time, the risk exists that DTs support a health financing scheme that has limited redistributive capacity and contributes to fragmentation and potentially to inequities in financing and access, such as medical savings accounts or voluntary health insurance for primary coverage. An example are mobile phone applications for collecting voluntary health insurance contributions or mobile health wallets used as individual saving accounts to make digital out-of-pocket payments [2], as found in Kenya and elsewhere [5].

**Figure 3:**
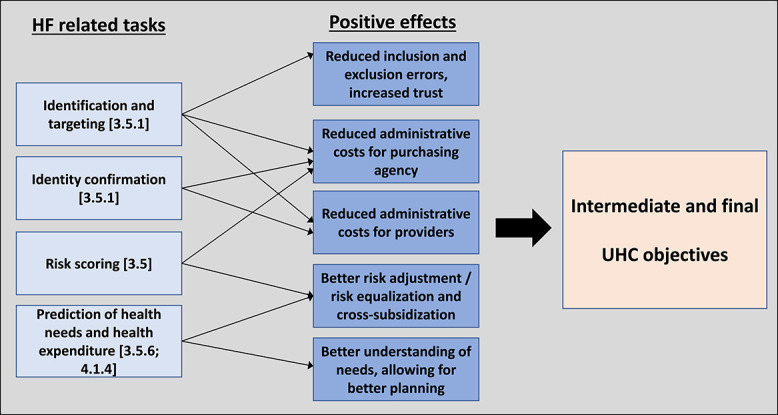
Pooling tasks supported by DTs, linked to potential positive effects and UHC objectives

Figure 3 summarizes the main tasks under the pooling function found to be supported by DTs and the potential positive effects, which may contribute to the intermediate and final UHC objectives.

### Purchasing

The use of DTs is found to be most prevalent in relation to the purchasing function. Among the numerous purchasing related tasks which can be enhanced or advanced through DTs, the ‘digitalization of claims (management) processes’ in health coverage schemes (such as health insurance programs) is probably the most fundamental step, which enables further advancements and innovations in other purchasing tasks. First and foremost, digital claims allow for the subsequent automation of claims submission, review and adjudication processes as well as payment of providers. Repetitive tasks of human reviewers can largely be eliminated through the use of pre-defined automated controls, leading to cost and time savings, easier identification of duplicate or flawed claims, thus decreasing erroneous payments, and reducing the time to pay providers. In Kenya for instance, the number of days taken to process claims from submission to payment reduced by almost one-third to a half. Similarly in Korea, digital claims management led to a reduction in the maximum review time from 40 days (for paper-based claims) to 15 days (for digital claims), while the payment time (i.e. the time between the confirmation that a claim has been reviewed and approved and the actual payment) reduced from 2 to 3 days to less than 1 day. In Estonia, the introduction of digital claims management resulted in a reduction of the total time needed for claims processing at the purchasing agency from 62 person-years in 2003 to 43 person-years in 2004. Efficiency gains can be even larger when claims are already automatically generated from electronic medical records at the level of the health service providers, reducing the administrative efforts at provider level, as is observed in North Macedonia (see Box 4).

**COUNTRY BOX 4: NORTH MACEDONIA**

Until 2013, the Health Insurance Fund (HIF) in North Macedonia had almost entirely relied on paper-based processes, resulting in weak coordination between the HIF branch offices, payment delays and a lack of unified data for decision-making. Under its Integrated Health Information Strategy (IHIS), first developed in 2006, the HIF has made great strides in the digitalization of its health information system. The system now supports the electronic contracting of health service providers and pharmacies (e-contracting), whereby contracts are signed digitally, contract data are captured in the information system of the HIF and part of this information is then made available online. Subsequently, it supports e-invoicing: monthly invoices are generated automatically from electronic health records and - after validation by an authorized representative of the health service provider - submitted to the web portal of the HIF. The availability of digital claims data has greatly simplified the claims management process for the HIF, as pre-defined automated controls can now be applied to check the provider contract conditions (and budget caps) as well as the insurance status of the patients, while also providing better data for decision making. Besides, there are also important efficiency gains: already after one year, the combined administrative savings for the HIF (less staff, less paper, less storage), for providers (idem) and for the population (less travel costs), were estimated to exceed the total initial investment by the HIF [13].

Yet, the effectiveness of digitalized claims management may be limited or its use even counter-productive if the design and implementation of this new business process is not sufficiently aligned with contextual conditions. For example, as experienced in Kenya, lack of human capacity and infrastructural limitations (such as lack of reliable internet) have hampered the use of digital claims management in lower-level public facilities. Many facilities continue with the cumbersome manual, paper-based claims management process while at the same trying to transition to digital claims management.

Another advanced purchasing task enabled by digital claims management is the automation of ‘claims review’ and ‘fraud detection’, which allows for more accurate detection of problematic claims at lower costs. Several countries, such as Estonia, the Republic of Korea and the Philippines (see Box 5), have started to test or adopt ML models to further enhance the detection of potentially fraudulent cases. While the number of use cases in practice is limited, there are numerous academic studies of modeling exercises published which test specific ML approaches for fraud detection in health insurance demonstrating their enhanced speed and accuracy [3]. As health service providers grow aware of these advanced detection techniques, purchasers expect more diligence in claims management.

**COUNTRY BOX 5: PHILIPPINES**

Based on the digital transmission of insurance claims from hospitals, the Philippine Health Insurance Corporation (PhilHealth) has explored and piloted the use of ML for fraud detection. In 2018, initial test findings demonstrated that a supervised algorithm could achieve a high accuracy in identifying providers which were suspected (or already known) to submit fraudulent or erroneous claims. Due to various reasons (including the complexity of the ML model and the lack of a sufficiently large and reliable training dataset for the supervised algorithm), from 2019 onwards the focus shifted to unsupervised ML techniques for outlier detection. Based on trends in claims submission and comparisons between doctors, the model identified providers exhibiting unusual claiming behavior for specific conditions, who were then subjected to investigation. While these measures led to an actual decrease in amounts claimed for the respective conditions, challenges existed in relation to data quality and to a lack of trained expert staff [16].

With the objective of moving toward more strategic purchasing, claims analysis can in turn also be used to inform the ‘setting of contracts’ between a purchaser and health service providers, including the ‘provider selection’ and the tailoring of contract conditions for each individual provider, such as a maximum number of cases (case volume) and available budget (budget volume) for each type of care, as is applied in Estonia (see Box 6). Another advanced task under the purchasing function enabled by digitized claims data is the ‘monitoring of contract execution’. For example, in North Macedonia and Estonia, contracts between purchasers and providers have been digitalized, as a result of which submitted claims can easily be checked against the agreed conditions and the status of contract execution (such as a comparison between the planned and the actual contract volume) can be monitored in near real-time. In comparison to paper-based claims, digitalized claims for health coverage schemes can of course also more easily be used to populate ‘business intelligence and visualization’ platforms, as practiced in Estonia. This enhances transparency and accountability since, for instance, the contract execution rates can be viewed by all providers.

**COUNTRY BOX 6: ESTONIA**

Between 2001 and 2004, the Estonian Health Insurance Fund (EHIF) established a uniform, centralized electronic channel (the **e-channel**), through which all health service providers submit their claims. In 2020, an upgraded version of the e-channel was introduced, which supports fully automated processing of claims, including the execution of pre-payment controls and the assignment of relevant claims to diagnosis-related groups (DRGs). The available digital claims data subsequently also inform the bi-annual setting of contracts with health providers, including provider-specific minimum and maximum contract volumes (number of cases) and budget caps per clinical specialty and per type of care. As the contracts themselves have also been digitalized, claims can then be checked in real-time against the contract volumes and conditions of the individual provider (contract monitoring) and the status of contract execution is updated on a daily basis. Deviations between planned and actual case volumes can subsequently inform the next round of contract setting, facilitating more strategic purchasing. Benefits of digital claims management include a reduction in errors and fraudulent claims, as well as cost-savings and a more equitable distribution of resources as a result of the new method of contract-setting [12].

Monitoring of provider behavior and performance in terms of quality of care can also be facilitated on the basis of digital claims, for instance by identifying under- or over-provision of specific health services or by checking the consistency between diagnosis and treatment. Such quality checks through in-depth claims analysis are for instance undertaken in Estonia, the Republic of Korea, North Macedonia and also explored in Kenya. In the Korean case, a three-step review process of claims subsequently also enabled digitalized pay-for-performance mechanisms, whereby financial incentives or penalties are directly linked to the outcomes of the reviews (see Box 7).

**COUNTRY BOX 7: REPUBLIC OF KOREA**

In the Republic of Korea, the National Health Insurance Service (NHIS) has been using a web-based claims submission portal since 2011. Digital claims undergo a three-step review process before payment: simple automated checks, a more detailed automated review against diagnosis-related criteria and an in-depth review of selected cases by human reviewers. Over the course of the years, several quality assessment and pay-for-performance initiatives have been introduced based on and using these digital claims data. For instance, under the Value Incentive Program, health facilities are incentivized or penalized on the basis of quality reviews. Among others, this has resulted in improved diabetes management and savings and a decrease in the prophylactic use of antibiotics during surgery and savings. A drug utilization review system is also in place, warning doctors and pharmacists in real-time in case of contraindications or otherwise dangerous drug use. As the NHIS is a single payer with mandatory health insurance (covering 97% of the Korean population), these improvements are nation-wide [14].

Moreover, digital claims data provide valuable information to feed into ‘health financing policy design processes and decision making for more strategic purchasing’. For example, digital claims data have been used to support the development of new provider payment methods (or their review and revision), in particular those that are data-intensive, such as risk-adjusted capitation payments, performance-based payments and case-based payment systems, as is the case in Estonia and the Republic of Korea. Likewise, DTs have enabled the ‘implementation of a more differentiated and needs-oriented coverage policy’ in Estonia to reduce co-payment spending on medicines. The e-prescription IT system, connecting multiple health data systems, now tracks how much a person has already spent on co-payments, on the basis of which it automatically calculates and determines in real-time a person’s eligibility for reduced co-payments on medicines for the remaining year. The benefit is then directly applied at the point of purchasing the medicine in the pharmacy, whereas in the past a person, if eligible, had to submit an application to the health insurance fund for the benefit to be calculated and reimbursed retrospectively. In the first year of implementation, this led to a 95% reduction in the number of people spending more than 250 euros per year on prescription drugs [20].

Almost without exception, these tasks can potentially be further supported in the future by ML, although with the risk that the use of incomplete or unrepresentative data could easily lead to unintended, distorted outcomes and unequal treatment of individuals, population groups or providers [3].

**Figure 4:**
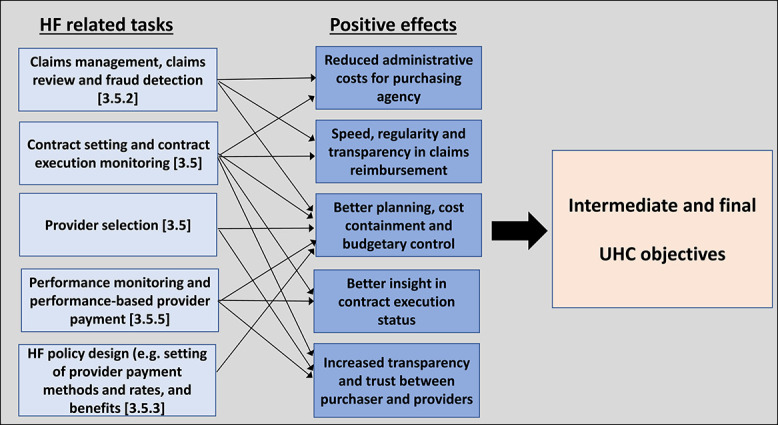
Purchasing tasks supported by DTs, linked to potential positive effects and UHC objectives

Figure 4 summarizes the main tasks under the purchasing function found to be supported by DTs and the potential positive effects, which may contribute to the intermediate and final UHC objectives.

## DISCUSSION

This synthesis reveals that DTs can positively affect health financing functions and tasks and thus contribute to achieving all intermediate UHC objectives. First and foremost, DTs contribute to enhancing efficiency and transparency. For instance, efficiency gains can be observed in revenue raising, as the time, effort and costs associated with making contributory payments are reduced. This resonates with other country experiences: findings from Nigeria suggest that digital health insurance management can help increase the number of enrollees [21]. Similarly, in various countries, digitalization of health insurance claims contributed to efficiency gains for both the purchaser (most notably through lower administrative costs, higher productivity and a reduction in erroneous payments) and health service providers [12, 14]. Digital claims data can also support a more equitable resource distribution, as they serve to inform provider contract setting in line with patient needs, or by enabling a more precise targeting and identification of poor and otherwise vulnerable population groups for subsidized health coverage.

We also see potential positive effects resulting from the emerging applications of AI and ML: efficiency, transparency and accountability could for instance benefit if the detection of fraud in insurance claims submission is supported by ML. Moreover, a more accurate prediction of health risks and expenditure could inform improved tailoring of services, leading to better quality of care and utilization in line with need [22].

The country studies showed that DTs help overcome fundamental challenges inherent in paper-based data systems and enable otherwise hardly implementable health financing policy options aimed at the final UHC objectives, especially those options that rely on near real-time exchange of data, such as the use of aggregate ceilings to individual out-of-pocket expenditure [20]. Likewise, more sophisticated stratification of contributions from people in the informal sector is hardly possible without a data architecture enabling near real-time updating of information.

Yet, the studies also point to various risks. Inequities can be exacerbated by digital divides, i.e. when certain population groups experience more difficulties in using and thus benefiting from DTs such as mobile phone applications, as observed in Kenya and Indonesia, similar to what is found for instance in Nigeria and Ghana [21, 23]. In addition, it is observed that the effects of the introduction of DTs may be sub-optimal due to a lack of human capacity within health financing institutions and/or due to infrastructural limitations, including unreliable Internet, as has been the case in Kenya as well as in Nigeria and Tanzania [17, 21, 24]. Particular caution is required when DTs enhance a pooling mechanism with limited redistributive capacity (e.g. mobile wallets for individual savings, without any inter-personal pooling function, or mobile phone applications for voluntary health insurance) [5].

The vigour of digitalizing health financing tasks also depends on a country’s health financing architecture. Five of the nine countries, purposively selected for the case studies due to their advanced practice, have a single-payer health system (a national health insurance system), and several had in fact defragmented their health financing system prior to starting a comprehensive digitalization process. Health financing reform and DT innovation can reinforce each other in a virtuous circle, as has been the case in the Republic of Korea, where the unification of several insurance schemes into the NHIS subsequently facilitated a comprehensive digital transformation, which in turn enabled new policies and reform initiatives, such as quality assurance and pay-for-performance schemes based on claims analysis [14]. This suggests that a national purchasing agency provides a larger potential for increased operational efficiency of the entire system through DTs. In a context of fragmented, multiple pools and payers, this may be more difficult both for practical and for political reasons: each purchaser will have to be convinced to adjust or abandon its existing system. Nonetheless, also in countries with multiple financing pools, DTs can be an effective vehicle and play a decisive role in improving coordination among those pools.

We observe that countries as different as Cambodia and Estonia have chosen to pursue step-by-step and phased incremental improvements as being more feasible than one big bang reform. Their digitalization approach has been tailored to their health financing arrangement as well as to the broader country context and capacity level, by carefully selecting the starting point for introducing the various DTs. In various countries (such as Rwanda and Kenya), digitalization of the beneficiary or membership management system was the entry point for digitalization, after which digital claims could be linked to the beneficiary data base. The digitalization of claims submission and management is often a strategic next step, as it creates many opportunities thereafter to optimize other purchasing-related tasks, as outlined above. Eventually, we see digitalization across the health financing functions, from revenue raising through pooling to purchasing, as is the case in Estonia, North Macedonia and the Republic of Korea and as is emergent in the other country studies.

Strategic sequencing of the digital transformation process is crucial; because once a first-order approach has proved to be successful, there can then be a path dependency that paves the way for gradual uptake of new applications, as is for instance observed in Cambodia [11]. The sequence of digitalizing health financing tasks may not always be intuitive: while, chronologically speaking, contract setting precedes contract monitoring, depending on the context it could in practice be more feasible to start using digital claims data for contract ‘monitoring’, as was the case in Estonia, before taking the more advanced step of deciding to ‘set’ contracts based on claims analysis, during which errors have more far-reaching consequences.

Importantly, several of the DTs under study required access to different data bases under the ownership of various agencies. Enabling and strengthening interoperability with information systems in use by other ministries and social protection programs therefore appear critical [5]. Eventually, interoperable data systems might even facilitate a fully automated process of pre-identification of beneficiaries [11].

This review has a number of limitations. The country case studies primarily used qualitative data, with some insights that could be gained through secondary data analysis. With some exceptions, however, it was difficult to get sufficiently detailed data to quantify and substantiate the effects of DTs on health financing. We also acknowledge that the group of countries studied was relatively small and rather diverse, including two high-income countries, two upper-middle income countries, four lower-middle income countries and one low-income country. The opportunities and challenges faced, in terms of digital infrastructure and ecosystem, resource envelope, stakeholder capacity and digital literacy inter alia, were thus also diverse. Moreover, the country case studies only assessed specifically selected DTs, instead of the full range of all DTs in use within one health coverage scheme. Studying all the DTs in use in health coverage schemes of several countries would have required a different study scope and scale.

## CONCLUSION: POLICY ORIENTATIONS AND SUGGESTIONS FOR FUTURE RESEARCH

We conclude this synthesis by deriving some policy orientations and suggestions for future research from the country study discussions, with the purpose of ensuring positive effects of DTs on health financing and UHC.

### Comprehensive approach: embedding health financing in national digital health strategies

To reap the benefits of DTs in health financing and minimize the risks, their introduction needs to be guided by a vision and a government strategy for digital transformation. This is to be reflected by national digital health strategies that pay explicit attention to DTs in health financing. This also needs to include a clear strategy on how the various existing digital divides within health financing can be addressed: between genders, urban and rural populations, higher- and lower-income groups, central and sub-national levels and between the public and the private sector.

### Regulatory frameworks: developing health financing specific regulations for DTs

There is need to develop health-financing specific regulations to ensure that DTs support UHC oriented health financing policy design and implementation. For example, safeguards are needed to avoid that DTs, for instance in the area of purchasing, are used to support cost reductions at the expense of quality of care or financial protection. Moreover, where DTs combine data from several databases, the country case studies pointed to the importance of adequate data governance to ensure data privacy, data security and data protection and to obtain people’s consent in relation to the use of DTs. Interoperability between data systems of various government agencies also requires clear data sharing policies, which specify the conditions for secure and responsible exchange of sensitive data. In relation to this, it is crucial to regulate which health financing data sets can be used and combined [25]. Clear stipulations are particularly required related to equity in DT use and outcomes, to make sure that the design of applications is user-centric and positive effects will also accrue to vulnerable population groups. Especially for DTs that rely on the access, knowledge, skills or financial resources of individual users, such as mobile phone applications, it is critical to monitor whether their introduction does not create or enhance inequities.

### The role of leapfrogging: adapting to context and capacity strengthening

Several of the country studies showed that the choice of context-appropriate DTs is also a matter of the available resources and the existing digital ecosystem in a country, for instance taking into account the share of the population that has regular access to the internet with an adequate connection. This, together with the lessons learnt from other countries already more advanced in applying DTs in health financing, can guide the right level for a country’s leapfrogging, whereby steps previously taken in other countries are skipped, and a more advanced technology is introduced right away. Such a more efficient and potentially shorter digitalization process may also bring down overall costs [12]. In all of this, it is also critical to ensure sufficient organizational and human-resource capacity among the health financing stakeholders, both in government and beyond. Only with sufficient(ly) qualified staff can the full benefits of DTs be reaped [12]. This may be even more true if the use of AI and ML is involved [16].

### Evidence generation: undertaking robust studies on the effects of DTs in health financing

For the future research agenda, more and methodologically robust evidence generation is needed, particularly through country case studies on low- and middle-income countries, in order to broaden our knowledge about the benefits and risks as well as other effects of the use of DTs for health financing tasks and functions. Pilots and scale up projects should be accompanied by continuous monitoring and evaluation during both the design and the implementation phase. The existing WHO guide with key questions and exemplary indicators to orient such country studies can help generate evidence on the effects of DTs in a systematic manner [10], with the ultimate objective of informing the design and implementation of DTs and optimizing the impact on health financing and UHC objectives. Future studies using this guide should be substantiated by more quantitative evidence of effects of DTs, making use of existing claims data and exploring other quantitative data sources. Given the rapid pace of innovation in this field, there is also a need for other types of knowledge generation, including action research and operational data analysis.

### Focus on the public interest: UHC driven research

Research on DTs for health financing must be based on—and driven by—a clear purpose in the public interest, system benefits and UHC objectives, rather than specific interests and gains of individual stakeholders [22]. Such UHC-oriented research as well as DT design would ideally be undertaken by multidisciplinary teams, involving health-financing policy experts as well as specialists from the relevant information technology, medical, legal and other domains to help formulate policy-relevant research questions and avoid negligence or underrepresentation of certain population groups [3].

### DTs as possible game-changers: being open to alternative health financing policy options

While DTs primarily serve to support health financing tasks, we need to consider the possibility that certain DTs may alter our thinking on health financing policy options, as they enable alternatives that—without digital means—would have been very cumbersome or impossible to implement. For instance, for the collection of contributions from the informal sector, mobile phone applications may prove to be a game-changer, a question that is to be explored through further research.

In conclusion, sound and tailored health-financing policy design remains the ultimate success factor for progress toward UHC, and DTs need to be designed and implemented in such a way that they can support and simplify health financing tasks. Every application of a DT for health financing requires a careful, context-specific assessment of the benefits and risks: whether the effects of the use of a DT will be positive or negative will mainly depend on how and for which purpose the DT is applied as well as on effective mitigation of the risks [3].

## Data Availability

The country reports with the data underlying this article will be shared on reasonable request by the corresponding author.
